# The Impact of Drought on HIV Care in Rural South Africa: An Interrupted Time Series Analysis

**DOI:** 10.1007/s10393-023-01647-6

**Published:** 2023-07-31

**Authors:** Collins C. Iwuji, Kathy Baisley, Molulaqhooa Linda Maoyi, Kingsley Orievulu, Lusanda Mazibuko, Sonja Ayeb-Karlsson, H. Manisha Yapa, Willem Hanekom, Kobus Herbst, Dominic Kniveton

**Affiliations:** 1 Africa Health Research Institute, Mtubatuba, KwaZulu-Natal South Africa; 2 Department of Global Health & Infection, Brighton and Sussex Medical School, University of Sussex, Falmer, Brighton, BN1 9PX UK; 3 Faculty of Epidemiology and Population Health, London School of Hygiene and Tropical Medicine, London, UK; 4 DSI-MRC South African Population Research Infrastructure Network (SAPRIN), Johannesburg, South Africa; 5 Centre for Africa-China Studies, University of Johannesburg, Johannesburg, South Africa; 6 Institute for Risk and Disaster Reduction, University College London, London, UK; 7 United Nations University Institute for Environment and Human Security, Bonn, Germany; 8 Kirby Institute for Infection and Immunity, University of New South Wales Sydney, Sydney, Australia; 9 Central Clinical School, University of Sydney, Sydney, Australia; 10 Division of Infection and Immunity, University College London, London, UK; 11 School of Global Studies, University of Sussex, Brighton, UK

**Keywords:** Drought, HIV, Adherence, Retention, Sub-Saharan Africa, South Africa

## Abstract

This analysis investigates the relationship between drought and antiretroviral treatment (ART) adherence and retention in HIV care in the Hlabisa sub-district, KwaZulu-Natal, South Africa. Data on drought and ART adherence and retention were collated for the study period 2010–2019. Drought was quantified using the 3-month Standard Precipitation Evapotranspiration Index (SPEI) and Standard Precipitation Index (SPI) from station data. Adherence, proxied by the Medication Possession Ratio (MPR), and retention data were obtained from the public ART programme database. MPR and retention were calculated from individuals aged 15–59 years who initiated ART between January 2010 and December 2018 and visited clinic through February 2019. Between 01 January 2010 and 31 December 2018, 40,714 individuals started ART in the sub-district and made 1,022,760 ART visits. The SPI showed that 2014–2016 were dry years, with partial recovery after 2016 in the wet years. In the period from 2010 to 2012, mean 6-month MPR increased from 0.85 in July 2010 to a high of 0.92 in December 2012. MPR then decreased steadily through 2013 and 2014 to 0.78 by December 2014. The mean proportion retained in care 6 months after starting ART showed similar trends to MPR, increasing from 86.9% in July 2010 to 91.4% in December 2012. Retention then decreased through 2013, with evidence of a pronounced drop in January 2014 when the odds of retention decreased by 30% (OR = 0.70, CI = 0.53–0.92, *P* = 0.01) relative to the end of 2013. Adherence and retention in care decreased during the drought years.

**Supplementary Information:**

The online version contains supplementary material available at 10.1007/s10393-023-01647-6.

## Introduction

Drought is one of the world’s most devastating climate hazards, particularly in regions with an annual rainfall of less than 500 mm (Ujeneza and Abiodun 2014). In Southern Africa, the annual rainfall of most areas is less than 500 mm and the occurrence of drought is often associated with food insecurity. Drought affects all four pillars of food security: availability, access, utilisation, and stability. For example, food availability is reduced by drought-related reductions in the productivity of crops, livestock, and fish (Mbow et al. 2019) (Lesk et al. 2016). These drops in productivity can also reduce access to food as the food purchasing power of households is reduced. In turn, food insecurity is associated with a range of poor health outcomes including increased risks of some birth defects, anaemia, cognitive problems, and aggression and anxiety (Berry et al. 2010; Gundersen and Ziliak 2015).

To date, only a few studies have investigated the impact of drought on HIV (Burke et al. 2015; Low et al. 2019, Austin et al. 2020). A study used data from 200,000 individuals from 19 countries to investigate the impact of local rainfall shocks on HIV prevalence. The authors found a statistically significant increase in infection rates in HIV-endemic rural areas by 11% for every recent drought. This was attributed to income shocks from the drought. However, this study did not investigate the impact of drought on HIV treatment and care (Burke et al. 2015). A study nested within the Lesotho Population-Based HIV Impact Assessment undertaken after the severe drought of 2014–2016 found an association between drought and higher HIV prevalence in young women in rural areas (Low et al. 2019). The study did not find an association between drought and awareness of HIV status, reported ART use and virologic suppression. However, this study had a few limitations; it was conducted after the drought and its cross-sectional design limited the ability to assign causality for observed associations. The drought was also very widespread with the majority of individuals (94%) living in drought-affected areas which could have limited the power of the study to address some of its objectives (Low et al. 2019). Another study concluded that drought was driving HIV transmission amongst vulnerable women in rural societies and proposed food insecurity as a mechanism (Austin et al. 2020).

While drought is only one of several factors affecting food security, we have also shown that it affects other non-food security factors that impact antiretroviral treatment (ART) adherence, such as migration and psychosocial issues disrupting interactions with health services (Orievulu et al. 2022a, b; Orievulu et al. 2022a, b). Nevertheless, multiple studies have shown an association between food insecurity and poor ART outcomes mediated through decreased adherence, poor absorption of antiretroviral drugs and economic constraints (Weiser et al. 2010; Bartelink et al. 2015; Iwuji et al. 2018).

In October 2014, KwaZulu-Natal declared a state of disaster due to the devastating effects of the El Niño-related drought being experienced across the country, with uMkhanyakude being one of the hardest-hit districts. Other provinces were also impacted by the drought with severity increasing in 2015 and expected to last until early 2016 culminating in many of the provinces declaring a state of disaster (South African Government 2015).

The consequent food shortages resulted in massive importation of food crops (like maize) to bridge resultant production shortfalls (Baudoin et al. 2017; Mare et al. 2018). This resulted in increase in the cost of food, further putting pressure on rural societies whose livelihoods were already precarious from pervasive poverty and the catastrophic costs of accessing care due to the high HIV and tuberculosis burden (Mudzengi et al. 2017).

South Africa has the largest global burden of HIV, with 7.8 million people living with HIV (PLHIV) in 2020 and the biggest ART programme (UNAIDS 2021). UNAIDS aims to eliminate HIV/AIDS as a public health threat by 2030 by ensuring 95% of PLHIV are diagnosed, 95% of those diagnosed on ART, and 95% of those on ART are virologically suppressed (UNAIDS 2014). Achieving these goals requires PLHIV to adhere lifelong to ART. In previous research, we developed a conceptual framework on how drought might affect the ability of PLHIV to adhere to treatment and remain in care (Orievulu et al. 2022a, b; Orievulu et al. 2022a, b).

Our study aims to investigate the impact of drought on ART adherence and retention in HIV care in rural Hlabisa, a sub-district in uMkhanyakude, KwaZulu-Natal. We hypothesise that the added shock from drought contributes to PLHIV prioritising livelihood sustenance over their health resulting in poor ART adherence and disruption in HIV care.

## Methods

### Study Design

We undertook an interrupted time series analysis (Bhaskaran et al. 2013; Bernal et al. 2017) of routinely collected data from 17 public sector primary care clinics in the Hlabisa sub-district, KwaZulu-Natal, South Africa. The Africa Health Research Institute (AHRI) through its population intervention programme has access to the national HIV care electronic patient records system (TIER.Net) used in all public sector clinics through a memorandum of understanding with the KwaZulu-Natal Provincial Department of Health.

### Study Setting

The Hlabisa sub-district is one of five sub-districts in the uMkhanyakude district municipality. The district has a population of 625,846 and is one of the most economically deprived districts in South Africa with extreme poverty; about 5% of adults have completed a higher education, 4% are covered by a medical aid scheme and unemployment rate is 62% (Gareta et al. 2021). About 53% of households are involved in agricultural activities compared to an average of 38% for the KwaZulu-Natal province (Let’s Respond). KwaZulu-Natal is the epicentre of the HIV epidemic in South Africa with prevalence among resident men and women aged 15–54 years in the Hlabisa sub-district in 2018 being 19% and 40%, respectively (Gareta et al. 2021). ART has been provided free of charge since the start of the HIV treatment programme in 2004, and TIER.Net was rolled out in 2010 (Osler et al. 2014).

### Participants and Procedures

Participants were individuals aged 15–59 years who were registered for HIV care and started ART in one of the 17 public ART clinics in the Hlabisa sub-districts between January 2010 and December 2018, with clinic visits through February 2019. TIER.Net has information on all HIV-positive individuals on ART including unique South African Identification number, age, sex, date of ART initiation, viral load results and dates, CD4 count results and dates, type of ART regimen, status of the individual (whether still in care, lost to follow up or dead), and dates of clinic attendance. A subset of these individuals were interviewed as part of a qualitative study to gain a further understanding of the economic, social and demographic impact of the drought on HIV treatment adherence with results published elsewhere (Orievulu et al. 2022a, b).

### Drought Quantification over Hlabisa Sub-District

The study analysed observational gridded and station datasets between 1995 and 2018. The observation data include monthly temperature and rainfall from the Climatic Research Unit Version 4.06 dataset (hereafter CRU) as is accessible from https://crudata.uea.ac.uk/cru/data/hrg/ (Harris et al. 2014). The CRU dataset covers all land areas (excluding Antarctica) at a 50 km resolution. The study also analysed monthly rainfall and temperature station data from the Riverview (32.1820°E; 28.4440°S) and Charters Creek (32.4140° E; 28.1970°S) rainfall weather stations, which were provided by the South African Weather Service. The CRU data were used to calculate drought over the entire Hlabisa sub-district (27.92°S–28.52°S; 32.79°E–32.62°E), while the station data were used to calculate drought near Riverview and Charters Creek which were the weather stations closest to the clinics and surrounding communities that contributed data to this analysis.

The 3-month Standard Precipitation Evapotranspiration Index (SPEI) (Vicente-Serrano et al. 2010) and Standard Precipitation Index (SPI) (McKee et al. 1993) were used to quantify drought over the Hlabisa sub-district for each of the observational datasets between 2010 and 2018. There were slight variations in drought estimates according to the data source and type of drought measurements used (supplementary appendix; Figure S1 & Figure S2).

### Outcomes

The primary outcomes for the current study were the ART medication possession ratio (MPR) during the 6-month interval after ART initiation, and retention in care 6 months after ART initiation. We calculated the MPR as [number of days with ART tablets]/[number of days in the interval]. For individuals who transferred to another clinic or died within the 6-month interval, we ended the interval on the day of transfer out or death. For all others, the interval ended 6 months after ART initiation, irrespective of whether the individual was attending clinic visits. Retention in care was assessed at 6 months after ART initiation (retained vs. not). Individuals who were recorded in TIER.Net as having transferred their care to another clinic were considered retained in care. Individuals who had not attended the clinic for > 3 months were considered lost to follow-up. For both outcomes, we used data from individuals who initiated ART between January 2010 and December 2018 and visit data through February 2019.

### Statistical Analysis

To summarise the data, we tabulated demographic characteristics and graphically depicted monthly trends in MPR and retention from 2010 to 2019. We then did an interrupted time series analysis of MPR by fitting a segmented linear regression model with Newey–West standard errors to account for autocorrelation and heteroskedasticity. We analysed retention using logistic regression with generalised estimating equations (GEE). For both analyses, we divided time into intervals chosen a priori based on the period of drought as reported by the South African government and allowed separate slopes and intercepts for each: (1) July 2010–December 2012; (2) January–December 2013; (3) January–December 2014; (4) January–December 2015; (5) January–December 2016; and (6) January 2017–February 2019. We used separate 1-year segments from 2013 to 2016, to allow the effect of drought on MPR and retention to vary from year to year and reduce the parametric assumptions over that period. We ran separate models stratified by sex, and by age group (15–24 years and 25–59 years) and adjusted for median CD4 count at ART initiation in year. We analysed data using Stata/IC version 17.0.

### Ethical Approval

This study was approved by the Biomedical Research Ethics Committee of the University of KwaZulu-Natal, South Africa (BREC Ref: BE004/19) and the Research Governance and Ethics Committee of the Brighton and Sussex Medical School (Ref: ER/BSMS9B5G/2).

## Results

### Characteristics of Study Participants

Between 01 January 2010 and 31 December 2018, 40,714 individuals started ART in the sub-district and made 1,022,760 ART visits by 28 February 2019 (Table 1). Their median (IQR) age was 30 (24–38) years, and 68% were women. Of the 40,714 individuals, over a third (37%) started ART in 2016–2018.

**Table 1:** Demographic characteristics of individuals starting ART at 17 clinics in Hlabisa, uMkhanyakude district.

	ART initiations *N* (col%)	ART visits *N* (col %)
*Year*		
2010–2012	11,711 (28.8%)	157,776 (15.4%)
2013–2015	13,892 (34.1%)	371,262 (36.3%)
2016–2018	15,111 (37.1%)	470,070 (46.0%)
Jan–Feb 2019	N/A	23,636 (2.3%)
*Age group*		
0–14	1919 (4.7%)	60,204 (6.0%)
15–24	8322 (20.4%)	112,855 (11.0%)
25–44	24,846 (61.0%)	640,962 (62.7%)
45–59	4768 (11.7%)	173,917 (17.0%)
≥ 60	858 (2.1%)	34,805 (3.4%)
Median (IQR) age	30 (24–38) years	34 (27–42) years
*Sex*		
Men	12,896 (31.7%)	305,583 (29.9%)
Women	27,818 (68.3%)	717,177 (70.1%)
Total	40,714	1,022,760

### Drought Trend in the Hlabisa Sub-District

**Figure 1:**
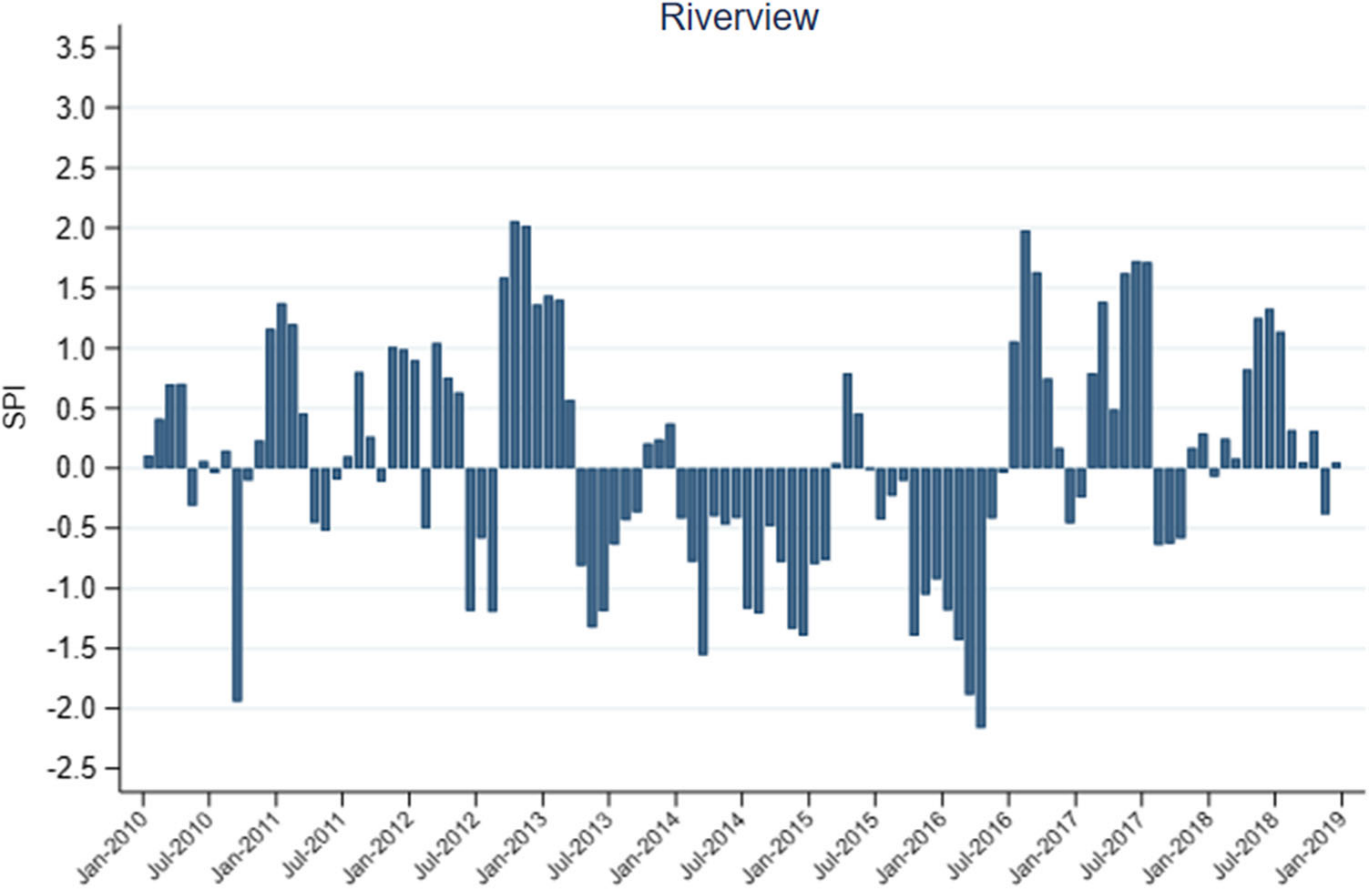
The interannual variability of the Standard Precipitation Index (SPI) measured using Riverview station data over the Hlabisa sub-district.

During the study period, all datasets showed similar agreement in SPEI and SPI droughts over the sub-district. For instance, most datasets showed drought between January and June 2010, while all datasets showed drought between 2014 and 2016, albeit with some discrepancies (supplementary appendix; Figure S1 & Figure S2). Although the observational datasets showed similar results in drought indices over the sub-district, the Riverview-SPI better captured droughts in Hlabisa as reported by the South African government (Fig. 1) (South African Government 2015). Given that, the results of this section will focus on the Riverview-SPI as an indicator for drought over the sub-district.

Figure 1 shows the interannual variability of Riverview-SPI droughts over the Hlabisa sub-district. The SPI shows abnormal wetness (+ ve SPI) for much of 2011, 2012 and early 2013 (Jan–Feb), neutral conditions in early 2014 and 2015, and drought conditions (−ve SPI) in mid-2013, much of 2014, late 2015 (Jul–Dec) and early 2016 (Jan–Jun). From July 2016 to 2018, SPI showed more wet conditions for Hlabisa.

### MPR and Retention in Care

**Figure 2:**
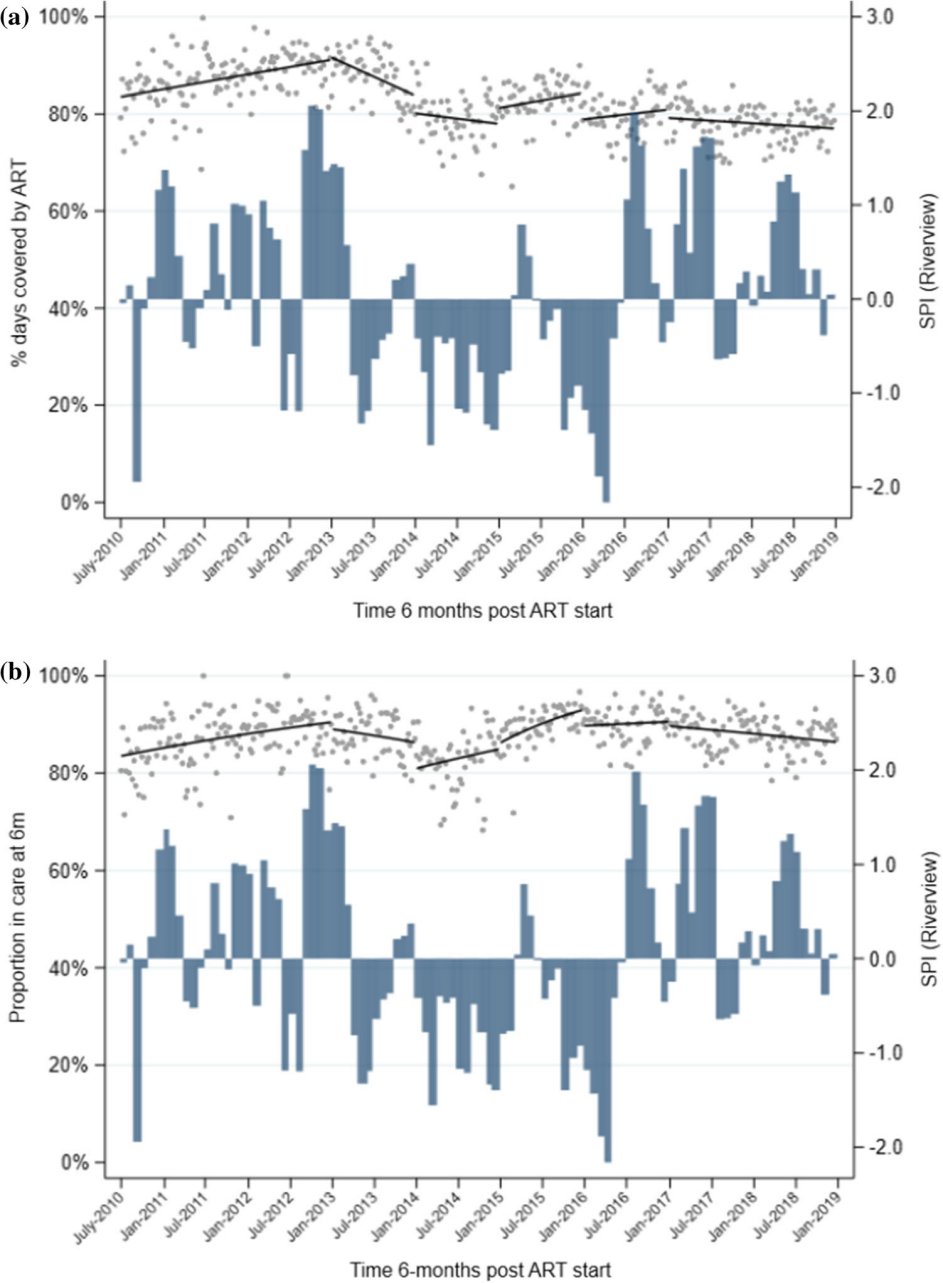
Overall medication possession ratio (MPR) during the first 6 months (**a**) and retention in care 6 months (**b**) after ART initiation at 17 clinics in Hlabisa sub-district, KwaZulu-Natal, from 2010 to 2019, with SPI from Riverview station data. **a** Scatterplot represents mean proportion of days covered for each week during the observation period; solid black lines represent the predicted values estimated by linear regression with Newey–West standard errors. **b** Scatterplot represents mean proportion retained each week during the observation period; solid black lines represent the predicted values estimated by GEE logistic regression.

**Figure 3:**
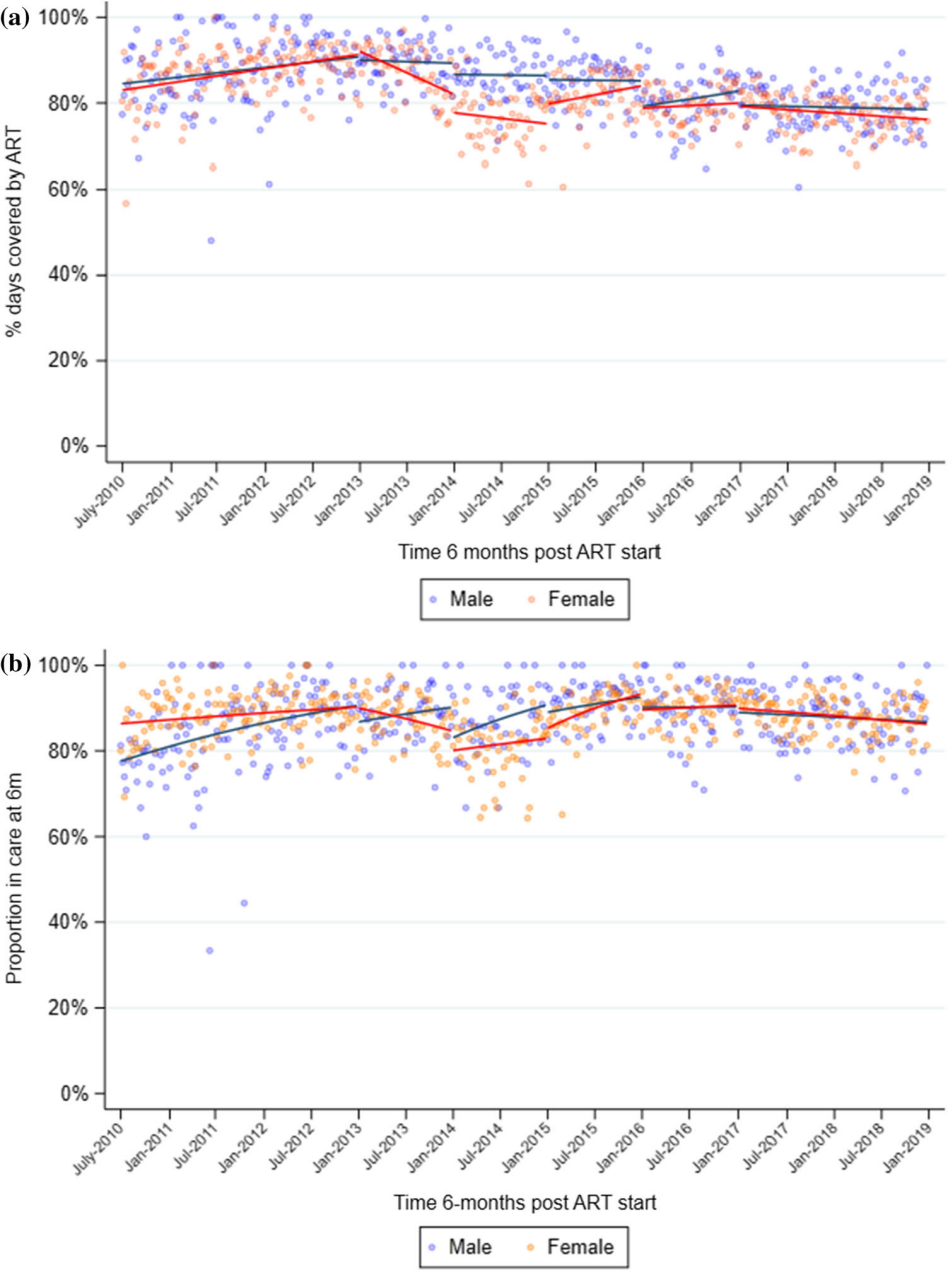
Medication possession ratio (MPR; **a**), and retention in care (**b**), 6 months after ART initiation at 17 clinics in Hlabisa sub-district, KwaZulu-Natal, from 2010 to 2019, stratified by sex. Scatterplot represents mean values each week during the observation period; solid lines represent the predicted values estimated by linear regression (MPR, **a**) and GEE logistic regression (retention, **b**).

**Figure 4:**
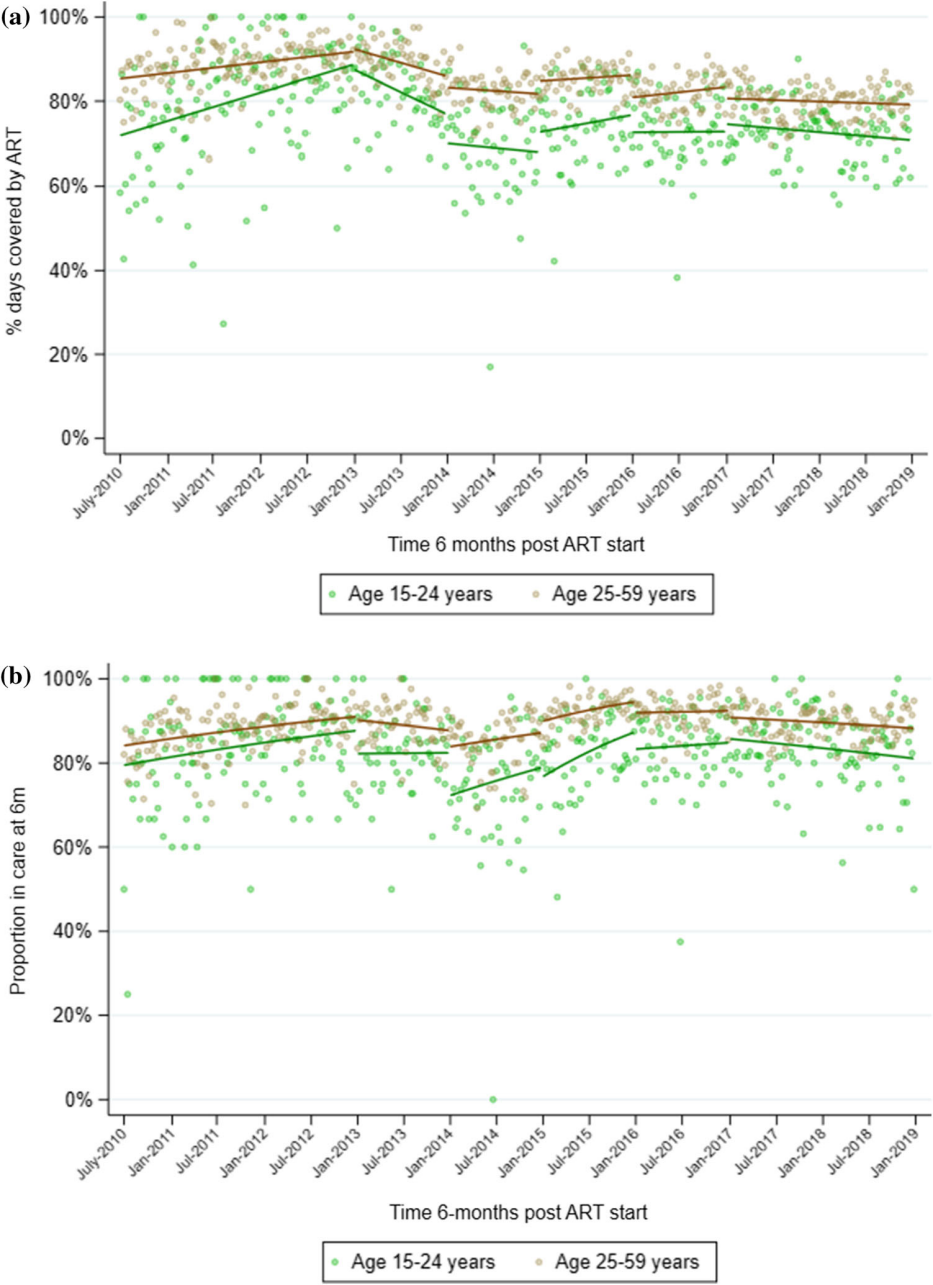
Medication possession ratio (MPR; **a**), and retention in care (**b**), 6 months after ART initiation at 17 clinics in Hlabisa sub-district, KwaZulu-Natal, from 2010 to 2019, stratified by age group. Scatterplot represents mean values each week during the observation period; solid lines represent the predicted values estimated by linear regression (MPR, **a**) and GEE logistic regression (retention, **b**).

In the period from 2010 to 2012, mean 6-month MPR increased from 0.85 in July 2010 to a high of 0.92 in December 2012 (Table 2, Fig. 2a). MPR then decreased steadily through 2013 and 2014, to 0.78 by December 2014. MPR began to increase again in 2015 to 0.84, but never reached the levels seen at the end of 2012, and instead remained between 0.78 and 0.80 through end 2018. Similar trends were seen when stratified by sex, although the decline in 2013 and drop at the start of 2014 was more pronounced among women (Table 2, Fig. 3a). When stratified by age, mean MPR in individuals aged 15–24 years was consistently lower than in individuals aged 25–59 years, although the trends (slopes) in each period were similar (Table 2, Fig. 4a). Younger individuals had a significant drop in mean MPR at the start of 2014, from 0.76 in December 2013 to 0.66 in January 2014.

**Table 2:** Segmented regression models of impact of drought on medication possession ratio (MPR) 6 months after ART initiation (regression coefficients and 95% confidence intervals^a^).

	Pre-drought	January–December 2013	January–December 2014	January–December 2015	January–December 2016	January 2017–December 2018
*All individuals*						
Mean MPR^b^	0.915	0.840	0.778	0.837	0.803	0.775
Trend^c^	0.001 (0.000,0.001)	−0.002 (−0.003,−0.000)	−0.001 (−0.002,0.001)	0.001 (−0.000,0.001)	0.000 (−0.000,0.001)	−0.000 (−0.001,0.000)
	*P* = 0.005	*P* = 0.006	*P* = 0.417	*P* = 0.231	*P* = 0.396	*P* = 0.139
Level change^d^	N/A	0.008 (−0.018,0.033)	−0.036 (−0.079,0.008)	0.034 (−0.005,0.073)	−0.053 (−0.086,−0.020)	−0.017 (−0.049,0.014)
		*P* = 0.562	*P* = 0.105	*P* = 0.091	*P* = 0.002	*P* = 0.287
*Men*						
Mean MPR^b^	0.909	0.893	0.863	0.848	0.825	0.787
Trend^c^	0.000 (−0.000,0.001)	−0.000 (−0.001,0.001)	−0.000 (−0.001,0.001)	−0.000 (−0.002,0.002)	0.001 (−0.001,0.002)	−0.000 (−0.001,0.000)
	*P* = 0.050	*P* = 0.693	*P* = 0.837	*P* = 0.882	*P* = 0.281	*P* = 0.635
Level change^d^	N/A	−0.007 (−0.036,0.023)	−0.026 (−0.056,0.005)	−0.009 (−0.052,0.034)	−0.060 (−0.111,−0.008)	−0.033 (−0.073,0.006)
		*P* = 0.653	*P* = 0.099	*P* = 0.688	*P* = 0.024	*P* = 0.100
*Women*						
Mean MPR^b^	0.915	0.821	0.750	0.837	0.797	0.773
Trend^c^	0.001 (0.000,0.001)	−0.002 (−0.003,−0.000)	−0.001 (−0.002,0.001)	0.001 (−0.000,0.002)	0.000 (−0.001,0.001)	−0.000 (−0.001,0.000)
	*P* = 0.019	*P* = 0.009	*P* = 0.474	*P* = 0.115	*P* = 0.644	*P* = 0.091
Level change^d^	N/A	0.010 (−0.026,0.046)	−0.043 (−0.095,0.009)	0.047 (−0.001,0.095)	−0.051 (−0.081,−0.020)	−0.007 (−0.039,0.025)
		*P* = 0.578	*P* = 0.105	*P* = 0.057	*P* = 0.001	*P* = 0.650
*Age 15–24 years*						
Mean MPR^b^	0.913	0.761	0.657	0.746	0.700	0.693
Trend^c^	0.001 (0.000,0.002)	−0.003 (−0.005,−0.000)	−0.001 (−0.003,0.001)	0.001 (−0.001,0.002)	−0.000 (−0.002,0.001)	−0.001 (−0.001,0.000)
	*P* = 0.017	*P* = 0.028	*P* = 0.422	*P* = 0.366	*P* = 0.858	*P* = 0.089
Level change^d^	N/A	−0.001 (−0.061,0.059)	−0.058 (−0.128,0.011)	0.052 (−0.019,0.123)	−0.039 (−0.102,0.025)	0.019 (−0.029,0.067)
		*P* = 0.974	*P* = 0.101	*P* = 0.153	*P* = 0.230	*P* = 0.436
*Age 25–59 years*						
Mean MPR^b^	0.923	0.861	0.814	0.852	0.825	0.790
Trend^c^	0.000 (0.000,0.001)	−0.001 (−0.002,−0.000)	−0.000 (−0.001,0.001)	0.000 (−0.001,0.001)	0.000 (−0.000,0.001)	−0.000 (−0.000,0.000)
	*P* = 0.023	*P* = 0.012	*P* = 0.458	*P* = 0.822	*P* = 0.240	*P* = 0.362
Level changed	N/A	0.008 (−0.016,0.033)	−0.025 (−0.066,0.016)	0.032 (−0.003,0.067)	−0.051 (−0.081,−0.022)	−0.027 (−0.059,0.004)
		*P* = 0.507	*P* = 0.228	*P* = 0.070	*P* = 0.001	*P* = 0.089

^a^Estimates from linear regression with Newey–West standard errors, adjusted for median CD4 count at ART initiation. ^b^Mean MPR at end of each period (December 2012; December 2013; December 2014; December 2015; December 2016; December 2018). ^c^Change in mean MPR from one month to the next. ^d^Immediate change in mean MPR at start of interval.

The mean proportion retained in care 6 months after starting ART showed similar trends to MPR, increasing from 86.9% in July 2010 to 91.4% in December 2012 (Table 3, Fig. 2b). Retention then decreased through 2013, with evidence of a pronounced drop in January 2014 when the odds of retention decreased by 30% (OR = 0.70, CI = 0.53–0.92, *P* = 0.01) relative to the end of 2013. As with MPR, retention began to increase again in 2015 and reached 89.5% by the end of 2016. When stratified by sex, the decrease in retention between 2012 and 2013 was greater among women than men, although the drop in January 2014 was smaller, with the odds of retention decreasing by 24% in women vs 45% in men (Table 3, Fig. 3b). When stratified by age, similar to MPR, mean retention was consistently lower in younger individuals than in older ones, but the trends over time were similar (Table 3, Fig. 4b). Among younger individuals, there was good evidence of a sizeable drop in retention in January 2014, when the odds of retention decreased by 39% (OR = 0.61, 95%CI = 0.42–0.90, *P* = 0.01) compared with the end of 2013.

**Table 3:** Segmented regression models of impact of drought on retention in care 6 months after ART initiation (regression coefficients and 95% confidence intervals^a^).

	Pre-drought	January–December 2013	January–December 2014	January–December 2015	January–December 2016	January 2017–January 2019
*All individuals*						
Mean retention^b^	91.4%	86.7%	84.3%	92.2%	89.5%	86.6%
Trend^c^	1.00 (1.00,1.01)	0.99 (0.98,1.00)	1.00 (1.00,1.01)	1.01 (1.00,1.02)	1.00 (1.00,1.01)	1.00 (0.99,1.00)
	*P* = 0.024	*P* = 0.092	*P* = 0.390	*P* = 0.002	*P* = 0.621	*P* = 0.002
Level change^d^	N/A	0.88 (0.68,1.14)	0.70 (0.53,0.92)	1.14 (0.85,1.54)	0.67 (0.52, 0.85)	0.90 (0.72,1.12)
		*P* = 0.331	*P* = 0.010	*P* = 0.382	*P* = 0.001	*P* = 0.347
*Men*						
Mean retention^b^	91.6%	90.4%	90.5%	91.1%	89.6%	86.1%
Trend^c^	1.01 (1.00,1.01)	1.00 (0.99,1.02)	1.01 (1.00,1.02)	1.00 (0.99,1.02)	1.00 (0.99,1.01)	1.00 (0.99,1.00)
	*P* = 0.002	*P* = 0.474	*P* = 0.060	*P* = 0.653	*P* = 0.705	*P* = 0.227
Level change^d^	N/A	0.69 (0.49,0.98)	0.55 (0.34,0.88)	0.87 (0.51,1.49)	0.76 (0.48, 1.19)	0.85 (0.57,1.26)
		*P* = 0.038	*P* = 0.013	*P* = 0.615	*P* = 0.227	*P* = 0.408
*Women*						
Mean retention^b^	91.5%	85.0%	82.3%	92.4%	89.3%	86.3%
Trend^c^	1.00 (1.00,1.01)	0.99 (0.98,1.00)	1.00 (0.99,1.01)	1.01 (1.00,1.03)	1.00 (0.99,1.01)	1.00 (0.99,1.00)
	*P* = 0.421	*P* = 0.039	*P* = 0.716	*P* = 0.007	*P* = 0.744	*P* = 0.004
Level change^d^	N/A	1.00 (0.70,1.43)	0.76 (0.54,1.05)	1.22 (0.85,1.75)	0.65 (0.47, 0.90)	0.93 (0.72,1.20)
		*P* = 0.987	*P* = 0.099	*P* = 0.281	*P* = 0.010	*P* = 0.563
*Age 15–24 years*						
Mean retention^b^	90.8%	82.4%	76.6%	85.9%	82.3%	80.0%
Trend^c^	1.00 (1.00,1.01)	0.99 (0.98,1.01)	1.00 (0.99,1.01)	1.01 (1.00,1.02)	1.00 (0.99,1.01)	1.00 (0.99,1.00)
	*P* = 0.396	*P* = 0.337	*P* = 0.665	*P* = 0.011	*P* = 0.907	*P* = 0.041
Level change^d^	N/A	0.71 (0.47,1.08)	0.61 (0.42,0.90)	0.91 (0.57,1.46)	0.74 (0.51, 1.08)	1.09 (0.78,1.54)
		*P* = 0.108	*P* = 0.012	*P* = 0.703	*P* = 0.120	*P* = 0.604
*Age 25–59 years*						
Mean retention^b^	91.4%	88.0%	87.0%	94.1%	91.9%	89.0%
Trend^c^	1.00 (1.00,1.01)	0.99 (0.98,1.00)	1.00 (1.00,1.01)	1.01 (1.00,1.02)	1.00 (0.99,1.01)	1.00 (0.99,1.00)
	*P* = 0.022	*P* = 0.279	*P* = 0.306	*P* = 0.034	*P* = 0.786	*P* = 0.035
Level change^c^	N/A	0.92 (0.67,1.27)	0.73 (0.53,1.02)	1.32 (0.95,1.83)	0.67 (0.49, 0.92)	0.82 (0.61,1.10)
		*P* = 0.611	*P* = 0.063	*P* = 0.094	*P* = 0.014	*P* = 0.180

^a^Estimates from GEE logistic regression, adjusted for median CD4 count at ART initiation. ^b^Mean % retained in care at end of each period (December 2012; December 2013; December 2014; December 2015; December 2016; December 2018). ^c^Odds ratio for the linear trend in being retained in care from one month to the next. ^d^Immediate change in odds of being retained at start of interval.

## Discussion

Our results show a marked decrease in ART adherence as measured by MPR during the drought years with no full recovery of adherence during the wet years. The decrease in adherence differed by sex and age with adherence being worse in women than men and in those aged between 15 and 24 years. While adherence in women returned to the level in men in Jan 2017 to Dec 2018, this recovery in adherence did not occur in younger individuals. There was also a marked drop in retention in care at the start of the drought years followed by some recovery and then, a dip again towards the end of the observation period. Similarly, the decrease in retention in care was more marked for women than men and in younger individuals than older individuals. These findings suggest that the dry spells in rural Hlabisa could have negatively impacted HIV treatment and care.

Drought years calculated from the Riverview-SPI, which typically coincide with El Nino events while wet years coincide with La Nina events in the Hlabisa subdistrict, are consistent with previous studies which found similar results over the Southern African region (Gore et al. 2020; Maoyi and Abiodun 2021, 2022). That is because El Nino years are characterised by anomalous upper tropospheric convergence (at 200 hPa) over the Southern African domain leading to increased subsidence and drought conditions, while La Nina years were characterised by anomalous upper tropospheric divergence (at 200 hPa) which may lead to enhanced convection and increased rainfall over the region (Maoyi and Abiodun 2021, 2022). The years 2014, 2015 and 2016 were particularly dry years in Hlabisa, as demonstrated by the SPI. Furthermore, these three years coincided with the 2014/2016 El Nino, which was the strongest ever recorded El Nino event since the 1997/1998 El Nino event (World Meteorological Organization 2015). On the other hand, the 2011–2012 SPI showed anomalous wetness, which is consistent with the La Nina event during those years.

Our key findings contribute to the plausible mechanisms by which drought may have resulted in a decrease in ART adherence and retention in care and draw attention to particularly vulnerable population groups such as women and youth. This is an interesting research finding confirming similar trends observed in another study which found increased suicide rates amongst women and youth during periods of extreme heat and humidity (Florido Ngu et al. 2021).

We hypothesise that drought may have exacerbated existing social vulnerabilities such as pervasive poverty, as well as patriarchal power relations and social barriers based in gender and age roles resulting in PLHIV prioritising livelihood sustenance over health-seeking behaviour. There is extensive literature describing how inherent gendered social structures particularly impact women and youth in times of environmental stress and shocks (Ayeb-Karlsson 2021; Florido Ngu et al. 2021). This manifested in the form of poor ART adherence and retention in care in this setting.

A recent study utilised data from 91 low-income countries to explore the direct and indirect influence of drought and food insecurity upon women's vulnerability to HIV. The study found that droughts are associated with HIV transmission among vulnerable women in poor countries and that food insecurity is a key mechanism in driving this relationship (Austin et al. 2020). Food insecurity has direct effects on HIV acquisition through macro- and micro-nutrient deficiencies with women being more likely to be food insecure than men (Friis 2005; Gillespie and Kadiyala 2005). This is often explained by gendered power relations leading women to eating less or skipping meals in periods of climatic stress to ensure that men, who often are the financial household providers, have sufficient amount of food. Indirectly, food insecurity negatively impacts women's socio-health status, including education, fertility, and access to medical care (Belachew et al. 2011; Hadley et al. 2012). Similarly, the unequal gender structure in low-income settings often pushes women and girls into child marriage, prostitution, transactional sex and other forms of risky sexual behaviour (Tsai and Weiser 2014; Pellowski et al. 2018; Masa et al. 2019).

As women are largely responsible for the household environment in low-income countries, climate-related environmental degradation such as drought could lead to increased difficulties in women securing access to food, drinking water, or other basic elements. Women are typically responsible for taking care of the household and children, as well as the bulk of the cooking, harvesting, and growing of food, and fetching water. This places a disproportionate burden on women who are sometimes socially punished or described as ‘less of a woman’ when under pressure from household chores during periods of climatic stress. Hence, we hypothesise that women were more likely to avoid, delay or wait longer to access health services (Gillespie and Kadiyala 2005; Hadley et al. 2012). Men are generally not as burdened by the household chores as they tend to work outside of the home, or manage agricultural and livestock resources that generate income (Agarwal 1997; Hovorka 2006). These differences in role between men and women may provide some explanations to our findings. However, studies in Africa that have examined retention in care in PLHIV outside the context of drought have found retention to be worse in men than women (Geng et al. 2010). Our findings that retention was worse in younger PLHIV is corroborated by another study in the same setting which investigated retention in individuals on ART during a period that included the drought years (Gosset et al. 2018). The relationship between age and adherence in South Africa was not consistent across studies with a few studies showing no association (Peltzer et al. 2010; Adakun et al. 2013; Iwuji et al. 2018).

Interviews carried out in a related qualitative study to inform the contextual reasons around our findings identified three interdependent themes elucidating the complex relationships between drought and HIV ART adherence namely; 1) economic (disrupted incomes, livelihoods and food systems), 2) social (water access, hygiene and sanitation challenges) and 3) demographic (human mobility) (Orievulu et al. 2022a, b). Suboptimal adherence and retention in care were often due to complex interacting socio-economic and demographic factors exacerbating existing social vulnerabilities such as poverty while eroding existing coping mechanisms. The drought resulted in food insecurity through loss of assets, crop or production failure and selling off assets due to disrupted incomes (Orievulu et al. 2022a, b).

For example, as explained by a woman who was not in care at the time of the interview.

*Because of the drought I am not able to eat and take my pills. However, when there is no drought*

*I am able to farm and … I take my vegetable, and make whatever I make, eat and drink my pills. [Woman, 49 years]*

About 53% of households in our study communities depend on agricultural activities for their livelihoods (Let’s Respond). Another study in the Hlabisa-sub-district done during the drought period found that over 40% of households in the sub-district were food insecure and being food insecure was associated with poor ART adherence (Iwuji et al. 2018). A Ugandan study showed that there is the belief that taking ART on an empty stomach will exacerbate side effects; hence, individuals skip their medications when there is no food.(Weiser et al. 2012) Our research findings reinforce impact of drought on food security and how this is linked to poor health outcomes (Berry et al. 2010; Gundersen and Ziliak 2015).

The drought increased the costs of food due to harvest failure (Mail and the Guardian 2016) while also resulting in a higher percentage of income being spent on food leaving little to pay for the transport costs to visit the clinics. Lack of transport fares has been shown to be associated with poor adherence and retention in care (Ehlers and Tshisuyi 2015; Adeniyi et al. 2018). This was confirmed by another woman who also was out of care at the time of the interviews undertaken in a prior qualitative study (Orievulu et al. 2022a, b).

*I missed (my) clinic appointment due to shortage of transport fare…. Let me put it like that. It happens that I am saving money to visit the clinic perhaps on the 20th of the month… [only] to find that money is not enough. Kids on the other side are complaining about bread. I then perhaps take this money and buy bread for them and say to myself the date … is still far. When the 20th comes, I have not been able to replace the money I took. As a result, I do not go to the clinic. *

*[Woman, 56 years].*

Our research participants, mostly women, described how they had to wake up early in the morning to travel long distances in search of water. On some occasions, they therefore had to miss their clinic appointments as they prioritised getting water for drinking and cooking. Other research studies have shown that people usually move their livestock to areas where the drought is less severe in periods of climatic stress (Bosongo et al. 2014; Eyassu et al. 2016; Nawrotzki et al. 2016). Migration away from usual residence has similarly also been shown to be associated with poor ART adherence and disengagement from care (Larmarange et al. 2018).

The strengths of our study include the use of a large dataset comprising over 40,000 PLHIV that allowed us to examine trends in ART adherence and retention in care over a long duration. We used an interrupted time series design, which is a robust and methodologically sound approach for evaluating the impact of exposures that cannot be randomised, in a real-world setting. We used the SPI to derive the segments for our regression model a priori and adjusted for CD4 counts at ART initiation to take account for changes in ART treatment guidelines and the evolution of the HIV epidemic. We also undertook a qualitative study to contextualise our findings (Orievulu et al. 2022a, b).

Our study had a few limitations. There may have been time-varying social or development-related factors that were associated with drought and potentially confounded the association with HIV care outcomes. We did not have data on these other factors or individual-level factors such as food insecurity, or a control group in another part of South Africa; however, one of the assumptions of interrupted time series analyses is that these factors would have changed relatively slowly over time at the population level and are partially accounted for by modelling the time trends (Bhaskaran et al. 2013; Bernal et al. 2017). Attributing impact to climatic changes is highly complex, and particularly when it involves human behaviour and responses. MPR is a proxy for adherence but cannot reveal whether an individual actually took their medication and may overestimate adherence (Lam and Fresco 2015). A hard outcome such as viral load suppression provides a better measure of adherence; however, our previous studies in this population have shown viral load monitoring to be sub-optimal and a large proportion of measurements are missing from TIER.Net (Iwuji et al. 2020). Additional limitations include missing data in TIER.Net owing to incomplete capturing of information, or transfers out to other facilities being misclassified as LTFU (Iwuji et al. 2022).

Extreme poverty, gendered inequalities, precarious labour, poorly maintained public water delivery systems, and general deprivation are some of the indicators that increased people’s vulnerability in uMkhanyakude (Massyn et al. 2020). Government policies that support investments in public health resources such as drinking water through installation of boreholes, availability of water trucks and food banks during periods of climatic stress would better enable people to cope and adapt. These response measures will only be successful, when involving the whole society and fully engaging local leaders and stakeholders to identify local drivers of vulnerabilities. The different government institutions responsible for drought management will need to collaborate more effectively to improve information/data sharing and early warning systems to facilitate planning and risk reduction leveraging both local knowledge and the technological strengths of institutions such as the South African Weather Service (Orievulu and Iwuji 2022).

Effective early warning and response system is critical for preparedness as the frequency of these weather extremes is predicted to increase due to climate change (Watts et al. 2021).

The findings from this interrupted time series analysis and the lived experiences of PLHIV in the sub-district suggest that during periods of drought, PLHIV are sometimes forced to prioritise their livelihood sustenance over their health which could impact their adherence to treatment and retention in care. Since this is the first study to our knowledge to investigate the impact of drought on ART adherence and retention in care, further studies involving larger geographical areas in South Africa and other sub-Saharan African countries with more varied precipitation, other social groups and alternative research designs that can better adjust for confounders are required to further our understanding of the dynamics in place.

### Supplementary Information

Below is the link to the electronic supplementary material.

Supplementary file1 (DOCX 4544 KB)

## Data Availability

The datasets generated during and/or analysed during the current study are available at the AHRI data repository, https://www.ahri.org/research/.

## References

[CR1] Adakun SA, Siedner MJ, Muzoora C, Haberer JE, Tsai AC, Hunt PW, Martin JN, Bangsberg DR (2013). Higher baseline CD4 cell count predicts treatment interruptions and persistent viremia in patients initiating ARVs in rural Uganda. J Acquir Immune Defic Syndr.

[CR2] Adeniyi OV, Ajayi AI, Ter Goon D, Owolabi EO, Eboh A, Lambert J (2018). Factors affecting adherence to antiretroviral therapy among pregnant women in the Eastern Cape, South Africa. BMC Infect Dis.

[CR3] Agarwal B (1997). "Bargaining" and Gender Relations: Within and Beyond the Household. Feminist Economics.

[CR4] Austin KF, Noble MD, Berndt VK (2020). Drying Climates and Gendered Suffering: Links Between Drought, Food Insecurity, and Women's HIV in Less-Developed Countries.* Soc Indic Res* 1–22 10.1007/s11205-020-02562-x PMC7685297 33250551

[CR5] Ayeb-Karlsson S (2021). ‘When we were children we had dreams, then we came to Dhaka to survive’: urban stories connecting loss of wellbeing, displacement and (im)mobility. Climate and Development.

[CR6] Bartelink IH, Savic RM, Dorsey G, Ruel T, Gingrich D, Scherpbier HJ, Capparelli E, Jullien V, Young SL, Achan J, Plenty A, Charlebois E, Kamya M, Havlir D, Aweeka F (2015). The effect of malnutrition on the pharmacokinetics and virologic outcomes of lopinavir, efavirenz and nevirapine in food insecure HIV-infected children in Tororo, Uganda. Pediatr Infect Dis J.

[CR7] Baudoin M-A, Vogel C, Nortje K, Naik M (2017). Living with drought in South Africa: lessons learnt from the recent El Niño drought period. International Journal of Disaster Risk Reduction.

[CR8] Belachew T, Hadley C, Lindstrom D, Gebremariam A, Lachat C, Kolsteren P (2011). Food insecurity, school absenteeism and educational attainment of adolescents in Jimma Zone Southwest Ethiopia: a longitudinal study. Nutrition Journal.

[CR9] Bernal JL, Cummins S, Gasparrini A (2017). Interrupted time series regression for the evaluation of public health interventions: a tutorial. Int J Epidemiol.

[CR10] Berry H, Bowen K, Kjellstrom T (2010). Climate change and mental health: A causal pathways framework. International Journal of Public Health.

[CR11] Bhaskaran K, Gasparrini A, Hajat S, Smeeth L, Armstrong B (2013). Time series regression studies in environmental epidemiology. Int J Epidemiol.

[CR12] Bosongo GB, Longo JN, Goldin J, Muamba VL (2014). Socioeconomic impacts of floods and droughts in the middle Zambezi river basin: Case of Kanyemba. International Journal of Climate Change Strategies and Management.

[CR13] Burke M, Gong E, Jones K (2015). Income Shocks and HIV in Africa. The Economic Journal.

[CR14] Ehlers VJ, Tshisuyi ET (2015). Adherence to antiretroviral treatment by adults in a rural area of Botswana. Curationis.

[CR15] Eyassu MA, Mothiba TM, Mbambo-Kekana NP (2016). Adherence to antiretroviral therapy among HIV and AIDS patients at the Kwa-Thema clinic in Gauteng Province, South Africa. African Journal of Primary Health Care & Family Medicine.

[CR16] Florido Ngu F, Kelman I, Chambers J, Ayeb-Karlsson S (2021). Correlating heatwaves and relative humidity with suicide (fatal intentional self-harm). Scientific Reports.

[CR17] Friis H (2005) Micronutrients and HIV infection: a review of current . World Health Organisation. Retrieved 30 Jan, 2022, from https://www.who.int/nutrition/topics/Paper%20Number%202%20-%20Micronutrients.pdf.

[CR18] Gareta D, Baisley K, Mngomezulu T, Smit T, Khoza T, Nxumalo S, Dreyer J, Dube S, Majozi N, Ording-Jesperson G, Ehlers E, Harling G, Shahmanesh M, Siedner M, Hanekom W, Herbst K (2021). Cohort Profile Update: Africa Centre Demographic Information System (ACDIS) and population-based HIV survey. Int J Epidemiol.

[CR19] Geng EH, Nash D, Kambugu A, Zhang Y, Braitstein P, Christopoulos KA, Muyindike W, Bwana MB, Yiannoutsos CT, Petersen ML, Martin JN (2010). Retention in Care among HIV-Infected Patients in Resource-Limited Settings: Emerging Insights and New Directions. Current HIV/AIDS Reports.

[CR20] Gillespie S, Kadiyala S (2005). HIV/AIDS and Food and Nutrition Security: Interactions and Response. American Journal of Agricultural Economics.

[CR21] Gore M, Abiodun BJ, Kucharski F (2020) Understanding the influence of ENSO patterns on drought over southern Africa using SPEEDY. Climate Dynamics **54**:307+.

[CR22] Gosset A, Protopopescu C, Larmarange J, Orne-Gliemann J, McGrath N, Pillay D, Dabis F, Iwuji C, Boyer S (2018) Retention in care trajectories of HIV-positive individuals participating in a universal test and treat programme in rural South Africa (ANRS 12249 TasP trial). *Journal of Acquired Immune Deficiency Syndromes* (1999). 10.1097/QAI.0000000000001938 PMC6410969 30570525

[CR23] Gundersen C, Ziliak JP (2015). Food Insecurity And Health Outcomes. Health Affairs.

[CR24] Hadley C, Stevenson EG, Tadesse Y, Belachew T (2012). Rapidly rising food prices and the experience of food insecurity in urban Ethiopia: impacts on health and well-being. Soc Sci Med.

[CR25] Harris I, Jones PD, Osborn TJ, Lister DH (2014). Updated high-resolution grids of monthly climatic observations—the CRU TS3.10 Dataset. International Journal of Climatology.

[CR26] Hovorka AJ (2006). The No. 1 Ladies' Poultry Farm: A feminist political ecology of urban agriculture in Botswana. Gender, Place & Culture.

[CR27] Iwuji C, Baisley K, Gareta D, Smit T, Siedner M, Herbst K (2022) Estimating retention in HIV care using data harmonization approaches: a longitudinal cohort study in rural KwaZulu-Natal, South Africa. *CROI Virtual*

[CR28] Iwuji C, McGrath N, Calmy A, Dabis F, Pillay D, Newell ML, Baisley K, Porter K (2018). Universal test and treat is not associated with sub-optimal antiretroviral therapy adherence in rural South Africa: the ANRS 12249 TasP trial. J Int AIDS Soc.

[CR29] Iwuji C, Shahmanesh M, Koole O, Herbst K, Pillay D, Siedner MJ, Baisley K, H.-D. Network (2020) Clinical outcomes after first-line HIV treatment failure in South Africa: the next cascade of care. *HIV Medicine*10.1111/hiv.12877 PMC7384088 32495515

[CR30] Lam WY, Fresco P (2015). Medication Adherence Measures: An Overview. Biomed Res Int.

[CR31] Larmarange J, Diallo MH, McGrath N, Iwuji C, Plazy M, Thiébaut R, Tanser F, Bärnighausen T, Pillay D, Dabis F, Orne-Gliemann J (2018) The impact of population dynamics on the population HIV care cascade: results from the ANRS 12249 Treatment as Prevention trial in rural KwaZulu-Natal (South Africa). *J Int AIDS Soc* 21 Suppl 4(Suppl Suppl 4):e25128 10.1002/jia2.25128 PMC6053480 30027600

[CR32] Lesk C, Rowhani P, Ramankutty N (2016). Influence of extreme weather disasters on global crop production. Nature.

[CR33] Let’s Respond. "A toolkit to integrating climate change risks and opportunities into municipal planning." Retrieved 19 Sept, 2021, from http://www.letsrespondtoolkit.org/municipalities/kwazulu-natal/umkhanuyakude-district-municipality---dc27

[CR34] Low AJ, Frederix K, McCracken S, Manyau S, Gummerson E, Radin E, Davia S, Longwe H, Ahmed N, Parekh B, Findley S, Schwitters A (2019). Association between severe drought and HIV prevention and care behaviors in Lesotho: A population-based survey 2016–2017. PLoS Med.

[CR35] Mail and the Guardian. (2016). "Full horror of drought emerges." Retrieved 27 Nov, 2022, from https://mg.co.za/article/2016-01-28-full-horror-of-drought-emerges/.

[CR36] Maoyi ML, Abiodun BJ (2021). How well does MPAS-atmosphere simulate the characteristics of the Botswana High?. Climate Dynamics.

[CR37] Maoyi ML, Abiodun BJ (2022). Investigating the response of the Botswana High to El Niño Southern Oscillation using a variable resolution global climate model. Theoretical and Applied Climatology.

[CR38] Mare F, Bahta YT, Van Niekerk W (2018). The impact of drought on commercial livestock farmers in South Africa. Development in Practice.

[CR39] Masa R, Graham L, Khan Z, Chowa G, Patel L (2019). Food insecurity, sexual risk taking, and sexual victimization in Ghanaian adolescents and young South African adults. Int J Public Health.

[CR40] Massyn N, Day C, Ndlovu N, Padayachee T (2020). District Health Barometer 2019/20.

[CR41] Mbow CC, Rosenzweig LG, Barioni TG, Benton M, Herrero M, Krishnapillai E, Liwenga P, Pradhan MG, Rivera-Ferre T, Sapkota FN, Tubiello Y, Xu (2019). Food Security. In:* Climate Change and Land: an IPCC special report on climate change, desertification, land degradation, sustainable land management, food security, and greenhouse gas fluxes in terrestrial ecosystems*, Shukla PR, Skea J, Calvo Buendia E, Masson-Delmotte V, Pörtner HO, Roberts DC, Zhai P, Slade R, Connors S, van Diemen R, Ferrat M, Haughey E, Luz S, Neogi S, Pathak M, Petzold J, Portugal Pereira J, Vyas P, Huntley E, Kissick K, Belkacemi M, Malley J (editors), In press.

[CR42] McKee TB, Doesken NJ, Kleist JR (1993) THE RELATIONSHIP OF DROUGHT FREQUENCY AND DURATION TO TIME SCALES.

[CR43] Mudzengi D, weeney S, Hippner P, Kufa T, Fielding K, Grant AD, Churchyard G, Vassall A (2017) The patient costs of care for those with TB and HIV: a cross-sectional study from South Africa.* Health Policy and Planning* 32(suppl_4):iv48–iv56. 10.1093/heapol/czw183 PMC5886108 28204500

[CR44] Nawrotzki RJ, Schlak AM, Kugler TA (2016). Climate, migration, and the local food security context: Introducing Terra Populus. Popul Environ.

[CR45] Orievulu K, Ayeb-Karlsson S, Ngwenya N, Ngema S, McGregor H, Adeagbo O, Siedner MJ, Hanekom W, Kniveton D, Seeley J, Iwuji C (2022). Economic, social and demographic impacts of drought on treatment adherence among people living with HIV in rural South Africa: A qualitative analysis. Climate Risk Management.

[CR46] Orievulu KS, Ayeb-Karlsson S, Ngema S, Baisley K, Tanser F, Ngwenya N, Seeley J, Hanekom W, Herbst K, Kniveton D, Iwuji CC (2022). Exploring linkages between drought and HIV treatment adherence in Africa: a systematic review. Lancet Planet Health.

[CR47] Orievulu KS, Iwuji CC (2022). Institutional Responses to Drought in a High HIV Prevalence Setting in Rural South Africa. International Journal of Environmental Research and Public Health.

[CR48] Osler M, Hilderbrand K, Hennessey C, Arendse J, Goemaere E, Ford N, Boulle A (2014). A three-tier framework for monitoring antiretroviral therapy in high HIV burden settings. J Int AIDS Soc.

[CR49] Pellowski JA, Huedo-Medina TB, Kalichman SC (2018). Food Insecurity, Substance Use, and Sexual Transmission Risk Behavior Among People Living with HIV: A Daily Level Analysis. Archives of Sexual Behavior.

[CR50] Peltzer K, Friend-du Preez N, Ramlagan S, Anderson J (2010). Antiretroviral treatment adherence among HIV patients in KwaZulu-Natal, South Africa. BMC Public Health.

[CR51] South African Government. (2015). "Government on water scarcity and drought." Retrieved 27 Nov, 2022, from https://www.gov.za/speeches/government-water-scarcity-and-drought-13-nov-2015-0000.

[CR52] Tsai AC, Weiser SD (2014). Population-based study of food insecurity and HIV transmission risk behaviors and symptoms of sexually transmitted infections among linked couples in Nepal. AIDS Behav.

[CR53] Ujeneza EL, Abiodun BJ (2014). Drought regimes in Southern Africa and how well GCMs simulate them. Climate Dynamics.

[CR54] UNAIDS. (2014). "FAST-TRACK. Ending the AIDS Epidemic by 2030." Retrieved 19/06/2018, 2018, from http://www.unaids.org/sites/default/files/media_asset/JC2686_WAD2014report_en.pdf.

[CR55] UNAIDS. (2021). "HIV estimates with uncertainty bounds 1990-Present." Retrieved 29 Jan, 2022,, from https://www.unaids.org/en/resources/documents/2021/HIV_estimates_with_uncertainty_bounds_1990-present.

[CR56] Vicente-Serrano S, Beguería S, López-Moreno JI (2010). A Multiscalar Drought Index Sensitive to Global Warming: The Standardized Precipitation Evapotranspiration Index. Journal of Climate.

[CR57] Watts N, Amann M, Arnell N, Ayeb-Karlsson S, Beagley J, Belesova K, Boykoff M, Byass P, Cai W, Campbell-Lendrum D, Capstick S, Chambers J, Coleman S, Dalin C, Daly M, Dasandi N, Dasgupta S, Davies M, Di Napoli C, Dominguez-Salas P, Drummond P, Dubrow R, Ebi KL, Eckelman M, Ekins P, Escobar LE, Georgeson L, Golder S, Grace D, Graham H, Haggar P, Hamilton I, Hartinger S, Hess J, Hsu SC, Hughes N, Jankin Mikhaylov S, Jimenez MP, Kelman I, Kennard H, Kiesewetter G, Kinney PL, Kjellstrom T, Kniveton D, Lampard P, Lemke B, Liu Y, Liu Z, Lott M, Lowe R, Martinez-Urtaza J, Maslin M, McAllister L, McGushin A, McMichael C, Milner J, Moradi-Lakeh M, Morrissey K, Munzert S, Murray KA, Neville T, Nilsson M, Sewe MO, Oreszczyn T, Otto M, Owfi F, Pearman O, Pencheon D, Quinn R, Rabbaniha M, Robinson E, Rocklov J, Romanello M, Semenza JC, Sherman J, Shi L, Springmann M, Tabatabaei M, Taylor J, Trinanes J, Shumake-Guillemot J, Vu B, Wilkinson P, Winning M, Gong P, Montgomery H, Costello A (2021). The 2020 report of The Lancet Countdown on health and climate change: responding to converging crises. Lancet.

[CR58] Weiser SD, Tsai AC, Gupta R, Frongillo EA, Kawuma A, Senkungu J, Hunt PW, Emenyonu NI, Mattson JE, Martin JN, Bangsberg DR (2012). Food insecurity is associated with morbidity and patterns of healthcare utilization among HIV-infected individuals in a resource-poor setting. Aids.

[CR59] Weiser SD, Tuller DM, Frongillo EA, Senkungu J, Mukiibi N, Bangsberg DR (2010). Food insecurity as a barrier to sustained antiretroviral therapy adherence in Uganda. PLoS One.

[CR60] World Meteorological Organization. (2015). "El Niño expected to be strongest since 1997–98." Retrieved 24 Feb, 2022, from https://public.wmo.int/en/media/news/el-ni%C3%B1o-expected-be-strongest-1997-98.

