# Correction: The Impact of Drought on HIV Care in Rural South Africa: An Interrupted Time Series Analysis

**DOI:** 10.1007/s10393-023-01648-5

**Published:** 2023-09-02

**Authors:** Collins C. Iwuji, Kathy Baisley, Molulaqhooa Linda Maoyi, Kingsley Orievulu, Lusanda Mazibuko, Sonja Ayeb-Karlsson, H. Manisha Yapa, Willem Hanekom, Kobus Herbst, Dominic Kniveton

**Affiliations:** 1 Africa Health Research Institute, Mtubatuba, KwaZulu-Natal South Africa; 2 Department of Global Health & Infection, Brighton and Sussex Medical School, University of Sussex, Falmer, Brighton, BN1 9PX UK; 3 Faculty of Epidemiology and Population Health, London School of Hygiene and Tropical Medicine, London, UK; 4 DSI-MRC South African Population Research Infrastructure Network (SAPRIN), Johannesburg, South Africa; 5 Centre for Africa-China Studies, University of Johannesburg, Johannesburg, South Africa; 6 Institute for Risk and Disaster Reduction, University College London, London, UK; 7 United Nations University Institute for Environment and Human Security, Bonn, Germany; 8 Kirby Institute for Infection and Immunity, University of New South Wales Sydney, Sydney, Australia; 9 Central Clinical School, University of Sydney, Sydney, Australia; 10 Division of Infection and Immunity, University College London, London, UK; 11 School of Global Studies, University of Sussex, Brighton, UK

**Correction to: EcoHealth** 10.1007/s10393-023-01647-6

In this article, the affiliation details of all authors were incorrectly given but should have been read as follows. The original article has been corrected.

