# Supplementary material for: The Impact of Drought on HIV Care in Rural South Africa: An Interrupted Time Series Analysis
Source: Ecohealth. 2023 Jul 31;20(2):178–93. doi: 10.1007/s10393-023-01647-6 (PMC10613144; doi:10.1007/s10393-023-01647-6)

**Figure S1** interannual variability of Standard Precipitation Evapotranspiration Index used to measure droughts using different data sources over the Hlabisa sub-district

**S1a** Riverview Station data


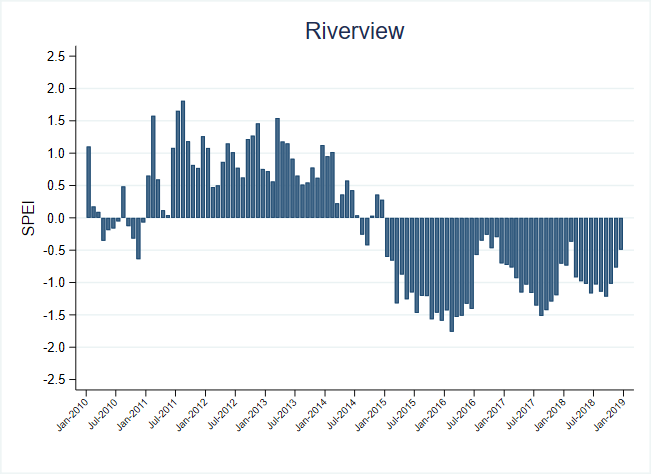


**S1b.** Charters Creek station data


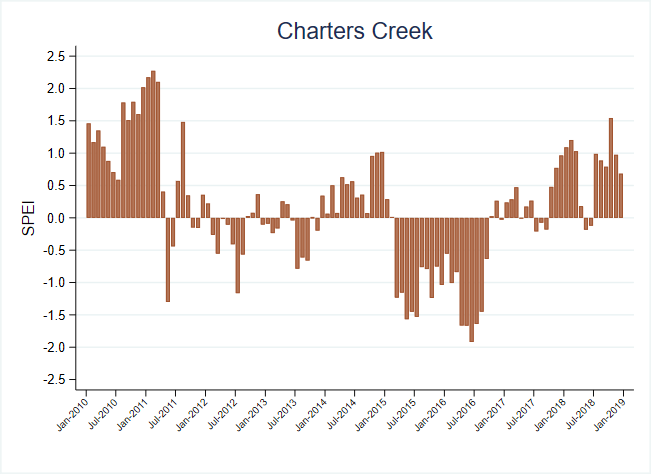


**S1c**. Climatic Research Unit


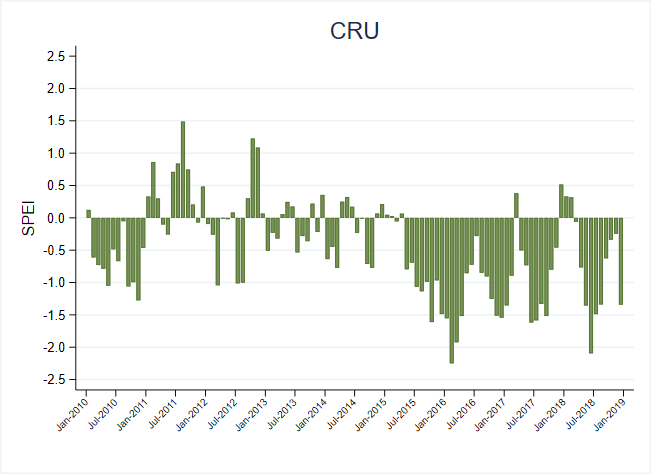


**Figure S2** interannual variability of Standard Precipitation Index used to measure droughts using different data sources over the Hlabisa sub-district

**S2a** Charters Creek


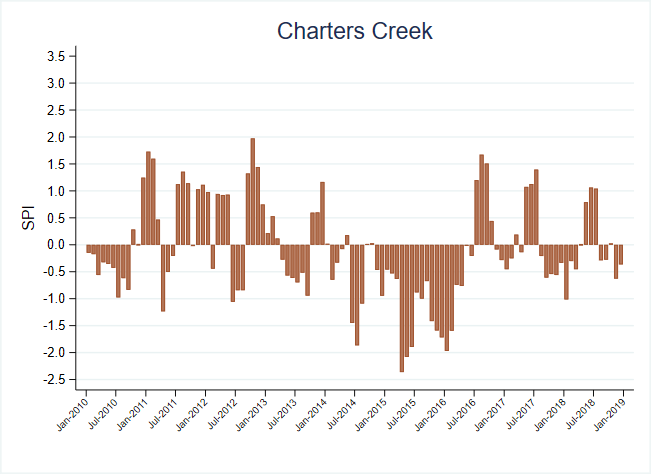


**S2b** Climatic Research Unit


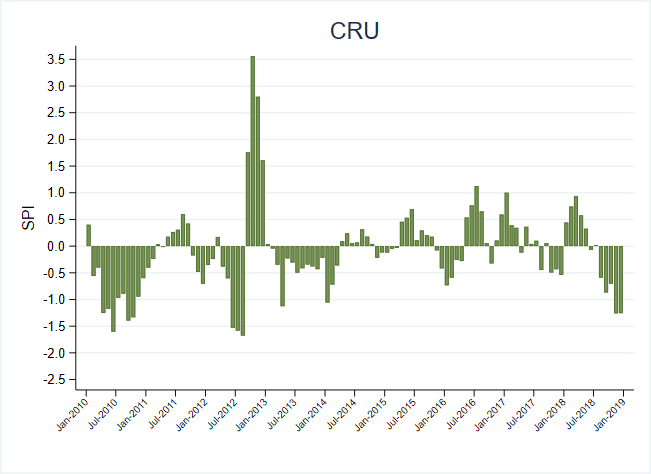

Supplement: Supplementary file 1 — Supplementary file1 (DOCX 4544 KB) [file 10393_2023_1647_MOESM1_ESM.docx]
